# Lorenz-63 Model as a Metaphor for Transient Complexity in Climate

**DOI:** 10.3390/e23080951

**Published:** 2021-07-25

**Authors:** Sergey Kravtsov, Anastasios A. Tsonis

**Affiliations:** 1 Atmospheric Science Group, Department of Mathematical Sciences, University of Wisconsin-Milwaukee, P.O. Box 413, Milwaukee, WI 53201, USA; aatsonis@uwm.edu; 2 Institute of Applied Physics, Russian Academy of Sciences, 603155 Nizhniy Novgorod, Russia; 3 Hydrologic Research Center, San Diiego, CA 92127, USA

**Keywords:** Lorenz model, climate, transient behavior

## Abstract

Dynamical systems like the one described by the three-variable Lorenz-63 model may serve as metaphors for complex natural systems such as climate systems. When these systems are perturbed by external forcing factors, they tend to relax back to their equilibrium conditions after the forcing has shut off. Here we investigate the behavior of such transients in the Lorenz-63 model by studying its trajectories initialized far away from the asymptotic attractor. Counterintuitively, these transient trajectories exhibit complex routes and, in particular, the sensitivity to initial conditions is akin to that of the asymptotic behavior on the attractor. Thus, similar extreme events may lead to widely different variations before the perturbed system returns back to its statistical equilibrium.

## 1. Introduction

The by now famous Lorenz-63 system [1] (hereafter, simply the Lorenz system or the Lorenz model), which arises via a truncation of Saltzman’s equations [2] for convective motion—a paramount feature in climate—is described by the following system of ordinary differential equations:

(1) $$\begin{array}{l} \dot{x}=-\sigma x+\sigma y,\\ \dot{y}=-xz+rx-hy,\\ \dot{z}=xy-bz. \end{array}$$

Here the dot denotes the time derivative, while the parameters σ and *r* correspond to the Rayleigh and Prandtl numbers, respectively. The choice of parameters σ=10, *r* = 28, *h* = 1, and *b* = 8/3 results in asymptotic (statistically equilibrated) aperiodic behavior on a strange attractor, with smooth trajectories alternating irregularly between loops around one of the two nontrivial unstable equilibrium points. In the original work by Lorenz, the parameter *h* in (1) was never varied; we introduced it here to aid a qualitative discussion of the transient behavior for the reasons that will become more apparent in Section 3. The topological structure and properties of the Lorenz attractor have been investigated and reported in a plethora of papers and books since the mid-1970s (see, for example, [3]).

Because of the great interest in the structural details of this—and other—chaotic attractors, their numerical simulations are usually initialized near the attractor itself. In this case the transients, defined as phase-space trajectories connecting the initial condition and the attractor, are short and uninteresting [4]. Less attention thus far was, however, paid to the transient behavior in situations when the Lorenz system is numerically integrated from the states located far from its asymptotic attractor. Investigating such transients is important because extreme far-from-equilibrium events do occur in nature due to either external forcing factors or due to self-amplifying interactions between various subcomponents of complex natural systems. Examples of the two types of phenomena in climate include the response of the climate system to forcing associated with volcanic aerosols and climate adjustment to particularly strong internal events associated with El Niño/Southern Oscillation, respectively; see [5] for a topical account of other important climatic interactions. The purpose of this note is to point out some interesting properties of post-extreme-event transients in the Lorenz model.

## 2. Duration of Transients and Its Relationship to Trajectory-Averaged Local Stability Multipliers

Asymptotically, as time t→∞, trajectories of the model (1) are confined within a bounded region *B* of the (*x*, *y*, *z*) phase space [5]. Here we objectively defined region *B* numerically, as a rectangular cuboid with *x-*, *y-*, and *z*-ranges based on maximum and minimum values of the corresponding variables from a long model simulation initialized on the attractor; these ranges are (–20, 20), (–27, 27), and (0, 48.5), respectively. We also defined the approximate center of the attractor as the long-term time mean of (*x*, *y*, *z*) from the same simulation: the point (0, 0, 24). We then performed numerical simulations of transient behavior in the Lorenz system (1) using a set of extreme initial conditions equidistant from the attractor center so computed and thus located on a sphere *S* with the radius *a* = 150; these initial conditions are all well beyond the attractor region *B*. The transients were defined as trajectories emanating from the sphere *S* and followed until their first entry into the region *B*. In most simulations, the system (1) was numerically integrated in time using the simplest Euler scheme with the time step Δt=0.001. In select cases, we confirmed our results with a much smaller time step of 0.0001 (not shown), which indicates that the dynamics we discuss here are not the artefacts of our numerical procedure.

**Figure 1:**
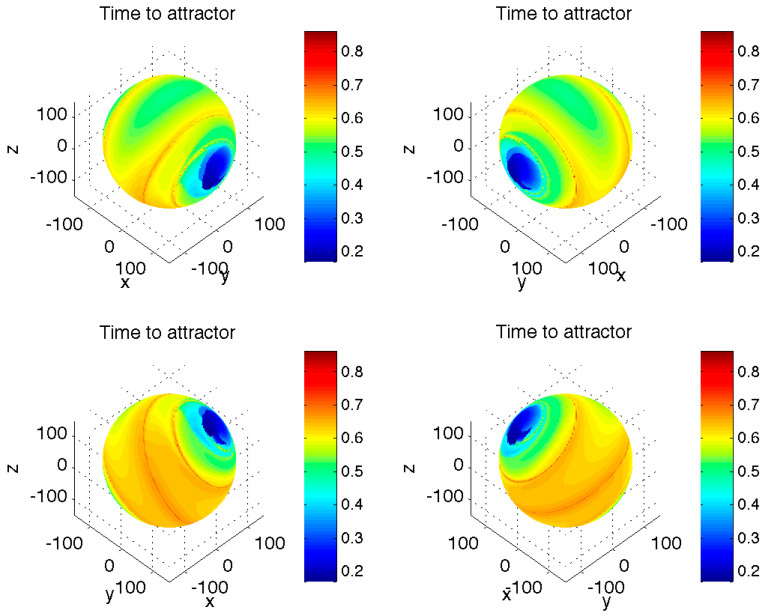
Distribution of transient times (color shading) to the Lorenz attractor for initial conditions on the sphere *S* with the radius *a* = 150 centered at the point (0, 0, 24); the center of this sphere was chosen to be close to the asymptotic time mean of trajectories simulated by the Lorenz model (1) with σ=10, *r* = 28, *h* = 1, and *b* = 8/3. The trajectory initialized on *S* was considered transient until its first entry into the rectangular cuboid region *B* bounded by *x*-, *y*- and *z*-ranges of (–19, 19), (–25, 25) and (4, 46), respectively. The Lorenz attractor for the model parameters considered is located within this region. The four figure panels display the same quantity, but from different view angles. Comment: *Note non-uniformity of the transient-time distribution, with a spiraling belt of relatively long durations*.

The first characteristic of the Lorenz-system transients we looked at was their duration (Figure 1) defined as the time it takes for a transient trajectory initialized on the sphere *S* to reach the region *B*. A typical time scale associated with a single revolution of a model trajectory on the butterfly-shaped asymptotic attractor about either lobe of this attractor for our choice of model parameters is on the order of unity (not shown). This also happens to be the duration of the longest transients for initial conditions on the sphere *S*; the fastest transients take as short as 0.2 time units to reach the attractor region *B*, while the mean duration of transients is around 0.6 time units. The most striking property of the transient times distribution is, however, its non-uniformity and, in particular, the presence of two “blue” regions of initial conditions leading to extremely short-duration transient trajectories, as well as the presence of a relatively narrow “red” spiral belt of the initial conditions corresponding to the trajectories with the longest transient-period durations.

We will see later that longer-duration transient trajectories are also the ones that exhibit the most interesting evolution. We found that a useful diagnostic for a potentially complex transient behavior can be obtained by computing the trajectory-averaged maximum local stability multipliers $\Lambda_{\max}$ [6] defined as the leading eigenvalue of the dynamical operator ℒ for the tangent-linear model that describes the local spread of trajectories of our original model (1) in the close neighborhood of an arbitrary point (*x*_0_, *y*_0_, *z*_0_) in the system’s phase space:

(2) $$\left(\begin{array}{c} \dot{\delta x}\\ \dot{\delta y}\\ \dot{\delta z} \end{array}\right)=ℒ\left(\begin{array}{c} \delta x\\ \delta y\\ \delta z \end{array}\right);\;ℒ=\left(\begin{array}{c} -\sigma \\ -z_{0}+r\\ -y_{0} \end{array}\;\begin{array}{c} \sigma \\ -1\\ x_{0} \end{array}\;\begin{array}{c} 0\\ -x_{0}\\ -b \end{array}\right)$$

**Figure 2:**
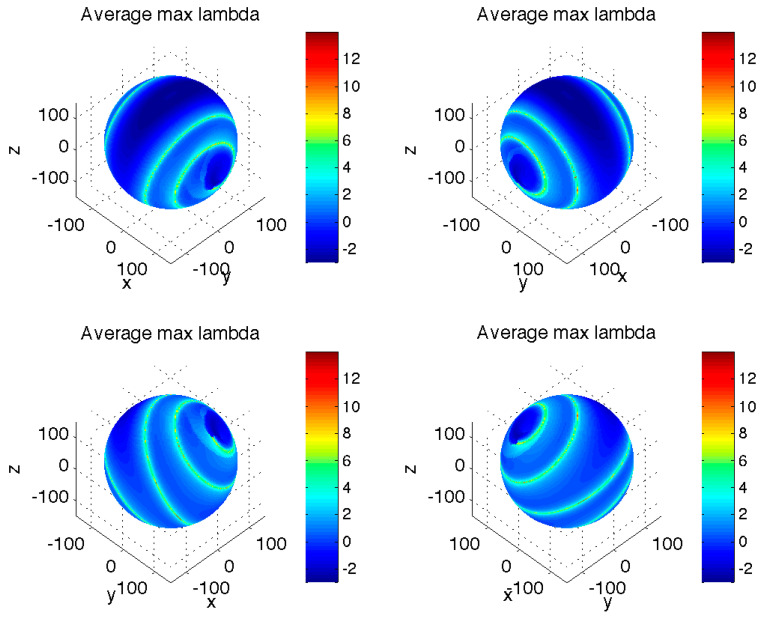
Distribution of the averaged maximum local stability multipliers $\Lambda_{\max}$ computed over the transient portion of trajectories (before first entry into the attractor region *B*) initialized on the same sphere *S* as in Figure 1. The same four orientations as in Figure 1 are shown. Comments: *Most of the trajectories exhibit positive local-stability-multiplier averages. There is a clear correspondence between the spiraling belt of largest averaged local stability multipliers and that of longest transient duration times in Figure 1*.

We computed $\Lambda_{\max}$ for all points along each transient trajectory between the sphere *S* and the asymptotic attractor region *B* and took an average of these values to characterize a given trajectory (Figure 2). Interestingly, a large fraction of initial conditions on the sphere *S* are characterized by the positive average local stability multipliers obtained, thereby indicating potential sensitivity to initial conditions, which we will indeed confirm in Section 3 below. Furthermore, there is a clear correspondence between the pattern of trajectory-averaged local stability multipliers in Figure 2 and that of the transient duration in Figure 1. In particular, the “blue” regions on the sphere *S* that initialize the transients with fastest decay toward the asymptotic attractor are also the regions with negative average local stability multipliers, while the “red” ribbon of initial states corresponding to the longest transients is also the region of the maximum positive trajectory-averaged local stability multipliers; using the finite-time Lyapunov exponents [7,8] (by estimating eigenvalues of the strain tensor *J*^T^*J*, where *J* is the finite-time Jacobian matrix whose evolution satisfies the tangent linear equations) to tag the trajectories produces essentially identical results (not shown). Thus, the longest transients are naturally associated with trajectories that tend to “travel sideways” along the dynamical slopes of the Lorenz-system “global” landscape, rather than going straight downhill toward the asymptotic attractor.

## 3. Types of Transient Behavior

### 3.1. Sensitivity of Transients to Initial Conditions

**Figure 3:**
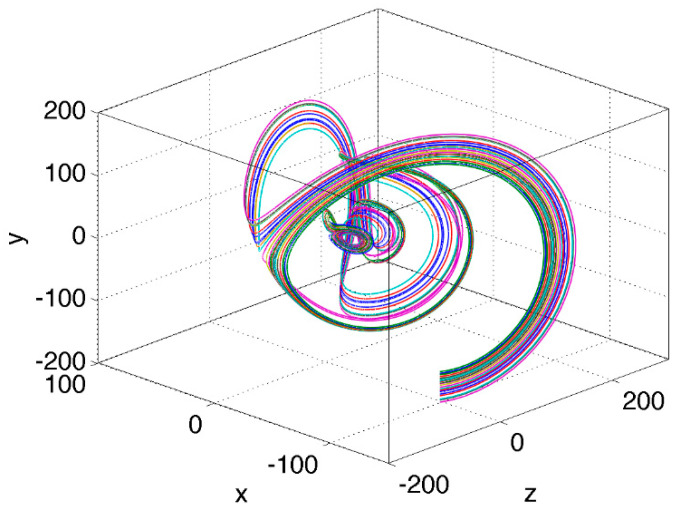
An example of transient trajectories sensitive to initial conditions. Comment: This is a typical situation for initial conditions taken in and around the spiraling belts of longest transient times (Figure 1) and highest averaged local stability multipliers (Figure 2).

A typical example of transient behavior for initial states chosen near the ribbon of longest transient times (Figure 1) or, equivalently, that of largest trajectory-averaged local stability multipliers (Figure 2), is shown in Figure 3. Here a bunch of trajectories that emanate from close-by initial conditions splits in two diverging sets of trajectories, which follow very different routes prior to reuniting near the asymptotic attractor location; the latter attractor region is evident as a small butterfly-shaped cluster of trajectories close to the origin. The immediate consequence of such transient behavior in the Lorenz system is that it can apparently be as unpredictable as the asymptotic behavior in the sense that similar extreme perturbations may result in completely unrelated transients as the system relaxes back to the state of its statistical equilibrium.

### 3.2. Geometric Complexity of Transients

**Figure 4:**
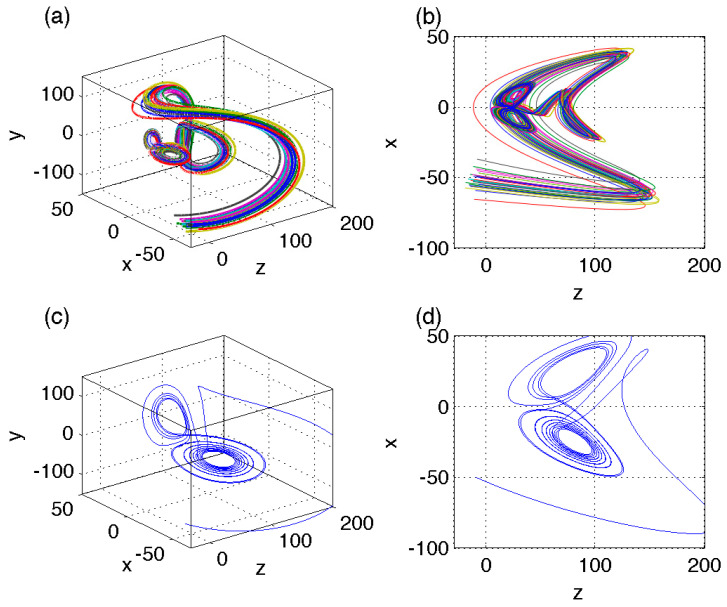
(**a**,**b**) An example of a “ghost” transient attractor in the simulation of the Lorenz model (1) with σ=10, *r* = 28, *h* = 1, and *b* = 8/3; (**c**,**d**) a trajectory of the Lorenz system with parameters (*σ*, *r*, *h*, *b*) stretched by *ε*^−1^ = 3. See text for details. Comments: *The transient trajectory in (a, b) does not simply spiral toward the attractor, but exhibits a complex path reminiscent of that on asymptotic attractor. The path of the rescaled Lorenz system in (c, d) shares geometrical similarity with the transient path in (a, b)*.

Another interesting observation is that transient approach to the asymptotic attractor may be characterized by fairly complex trajectories, which appear, in some cases, to emulate the attractor itself (Figure 4a,b). In particular, the trajectories here exhibit larger-scale butterfly-shaped excursions prior to ending up on the similarly shaped asymptotic attractor near the origin. We will refer to this phenomenon as to the “ghost” transient attractor, with the intentionally oxymoronic character of the term implying the presence of a temporary geometrical structure lurking in the phase-space region far away from the statistically equilibrated long-time asymptotic solution.

Qualitatively, this behavior can be understood in the following way. We saw previously that some transients are able to stay away from the attractor for a longer time than others (Figure 1). For such longer transient trajectories, the variables in the model (1) can be locally rescaled in space and time to focus on their relatively persistent (large) local phase-space distances from the origin and fast phase speeds. Effectively, this rescaling will produce a system of equations completely analogous to Equation (1), but with different set of parameters (*σ*, *r*, *h*, *b*). If so, it may not seem improbable that for some regions of the phase space, this “transient” Lorenz model will exhibit dynamical structures and trajectory shapes akin to those known to arise for other parameter sets in the asymptotic limit of statistically equilibrated behavior.

To present a concrete example of the rescaling mentioned above, we introduce the following change of variables

(3) $$(x^{\prime },y^{\prime },z^{\prime },t)=\varepsilon (x,y,z,t^{\prime }).$$

Note that for ε<1, (3) corresponds to squishing the spatial coordinates and stretching the time so that the large values of the non-transformed variables on the order of $\varepsilon^{-1}$ will correspond to the transformed variable values on the order of one, while the order-of-one changes in the stretched time $t^{\prime }$ will span the short interval on the order of *ε* when measured in original time units *t*. This rescaling is thus appropriate, in principle, for the trajectories in the region situated far from the origin and during a relatively short transient period before returning to the asymptotic attractor behavior.

Substituting the transformation (3) into the system (1) and introducing the new set of parameters

(4) $$\left(\sigma^{\prime },r^{\prime },h^{\prime },b^{\prime })=\varepsilon (\sigma ,r,h,b\right)$$

results, for this case, in the *same* system of equations as the original system (1), but for the primed variables. This implies that topological behavior of the trajectories that are somehow *able to persist* in a far-away-from-attractor region of the phase space in which (3) is valid can be qualitatively described by the asymptotic behavior of the Lorenz system with a different set of parameters rescaled by $\varepsilon^{-1}$, where *ε* corresponds to the ratio of the radius-vector of defining the far-away location of the persistence region to the typical value of the trajectories’ radius-vector in the original Lorenz system. Indeed, the topology of the Lorenz system (1) with (σ,r,h,b) parameters rescaled in this way using the value of ε=1/3 (Figure 4c,d) looks qualitatively similar to the transient trajectories in Figure 4a,b.

Note that in the rescaling example (3), (4), the notion of the single parameter *ε* controlling the stretching of all three phase-space variables, as well as time, is completely arbitrary. Furthermore, and more importantly, the local stretching (3) tells nothing about where in the phase space the transient trajectories must be for the stretched regime to be persistent; the latter persistence is essential for these trajectories to have sufficient time to reveal the structure of the stretched-system attractor during transient evolution. For these reasons, the qualitative demonstration above should be regarded as nothing more as an empirical one-parameter fit to illustrate the concept of the “ghost” transient attractor.

## 4. Summary and Discussion

We studied transient behavior in numerical simulations of the three-variable Lorenz model (1) initialized far away from the region of its asymptotic chaotic attractor. These transients were shown to have a range of durations, with the longest transients corresponding to the trajectories having largest average local stability multipliers and complex routes emulating sensitivity to initial conditions, as well as exhibiting the “ghost” attractors akin to their asymptotic siblings.

Persistent chaotic transients in the Lorenz system have been studied before in the particular case when the Rayleigh number was chosen to be just below the critical value required for chaotic behavior [9,10]; this regime has been dubbed the pre-turbulence regime [11]. With this choice of parameters, the model trajectories initially evolved along the attractor that was close to the asymptotic chaotic attractor of the system with a slightly higher Rayleigh number, but slowly decayed from chaos to the final state of a steady flow. This situation is different from the one considered in the present paper, where the parameters of the Lorenz model were set to correspond to the chaotic regime; the non-trivial transients arise here due to the dynamical properties of the system considered in the phase space regions situated far from the asymptotic attractor.

A recent study of pre-turbulence in the Lorenz system [12] elucidated an important role of global invariant manifolds in defining the topological structure of long transients in this ‘pre-chaotic’ regime. Interestingly, for the classical chaotic regime considered here, the locations (on our chosen sphere) of the initial conditions that result in the longest transients (Figure 1 and Figure 2) are also apparently connected to the geometry of the Lorenz manifold [13]—the stable manifold of the origin (compare our Figure 1 and Figure 2 with Figure 1 of that paper and its animated online version). The global structure of the Lorenz manifold, which dictates asymptotic destiny of the trajectories started at an arbitrary initial condition, is indeed intricate [14]. Our results, however, emphasize a different type of transient complexity: for example, temporary divergence of initially close transient trajectories (and thus their widely different routes) far before they reach the asymptotic attractor, as well as their ‘ghost attractor’ behavior. Further studies are necessary to examine in detail the role of the Lorenz manifold in the transient dynamics discussed here.

Transient behavior of dynamical systems recently drew a lot of attention in the ecological literature (see [15] and references therein). The discussion in [15] evolved around recognizing the fact that the transient behavior is closely associated with the inherently multi-scale character of natural systems, including the timescale asymmetries stemming from the presence of the stable and unstable manifolds in these systems’ dynamics; incidentally, this presence is the root of the strange chaotic attractor in the Lorenz model. Cushing et al. [16] described laboratory experiments and numerical simulations of the transient behavior in an underlying population model, which depended on the choice of the initial conditions near the stable or, alternatively, unstable manifold of an equilibrium point of this model. This sensitivity of the transient evolution to initial conditions is qualitatively similar to the behavior we report here, but involves completely different dynamics, which lack, for example, the “ghost-attractor” behavior.

The properties of the transient behavior in the Lorenz model discussed here are not just beautiful but may also have important implications in understanding the evolution of complex nonlinear systems such as climate, economy, ecosystems, sociological networks and so on, if these systems are somehow taken far from their equilibrium states. In particular, similar extreme perturbations in such systems may exhibit widely different variations before relaxing back to the statistical equilibrium.

## Data Availability

Data is available from the authors.
